# Sequence‐Similar Protein Domain Pairs With Structural or Topological Dissimilarity

**DOI:** 10.1002/prot.26753

**Published:** 2024-10-11

**Authors:** Peter Røgen

**Affiliations:** ^1^ Department of Applied Mathematics and Computer Science Technical University of Denmark Kongens Lyngby Denmark

**Keywords:** chain topology, metric on sequence alignments, nonredundant, structure alignment, structure comparison

## Abstract

For a variety of applications, protein structures are clustered by sequence similarity, and sequence‐redundant structures are disregarded. Sequence‐similar chains are likely to have similar structures, but significant structural variation, as measured with RMSD, has been documented for sequence‐similar chains and found usually to have a functional explanation. Moving two neighboring stretches of backbone through each other may change the chain topology and alter possible folding paths. The size of this motion is compatible to a variation in a flexible loop. We search and find domains with alternate chain topology in CATH4.2 sequence families relatively independent of sequence identity and of structural similarity as measured by RMSD. Structural, topological, and functional representative sets should therefore keep sequence‐similar domains not just with structural variation but also with topological variation. We present BCAlign that finds Alignment and superposition of protein Backbone Curves by optimizing a user chosen convex combination of structural derivation and derivation between the structure‐based sequence alignment and an input sequence alignment. Steric and topological obstructions from deforming a curve into an aligned curve are then found by a previously developed algorithm. For highly sequence‐similar domains, sequence‐based structural alignment better represents the chains motion and generally reveals larger structural and topological variation than structure‐based does. Fold‐switching protein pairs have been reported to be most frequent between X‐ray and NMR structures and estimated to be underrepresented in the PDB as the alternate configuration is harder to resolve. Here we similarly find chain topology most frequently altered between X‐ray and NMR structures.

## 1 Introduction

**FIGURE 1:**
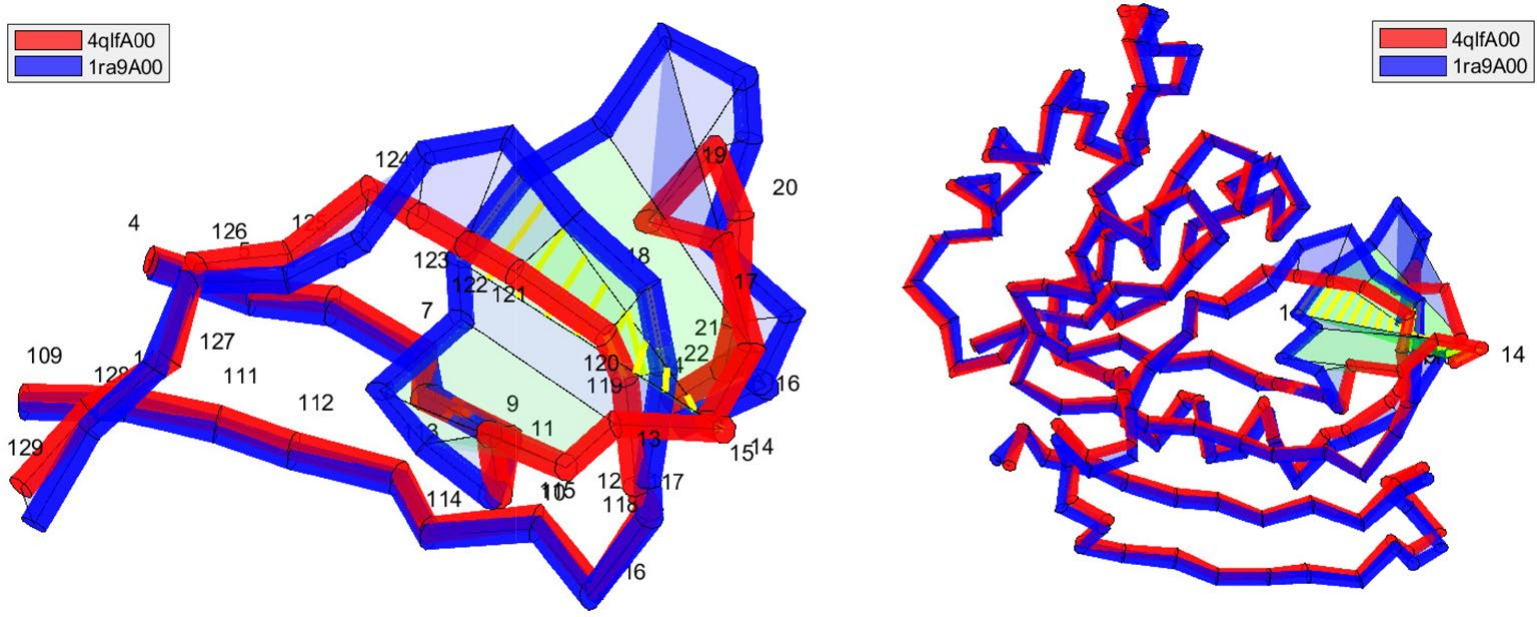
4qlfA00 and 1ra9A00 have 99% sequence identity and both sequence‐based and structure‐based gap‐free RMSD≈2.5 Å. The linear interpolation between the aligned backbone curves is shown as a surface. The yellow line segments show where the backbone passes through itself. 1ra9A pokes through an open loop whereas 4qlfA passes outside that loop. An end‐contraction of 14 residues can resolve this morph self‐intersection but it removes re‐threads and puts back one of the central strands in the beta sheet. Right: a zoom. The numbers refer to the (pseudo) alpha carbons of the curve alignment explained in the text.

Kosloff and Kolodny [1] reported sequence‐similar, structure‐dissimilar protein chain pairs in the PDB. Here we at the domain level similarly find sequence‐similar RMSD‐dissimilar CATH4.2 domains, but our main focus is to search for sequence‐similar pairs with distinct chain topology. We start with the example shown in Figure 1. Structural alignment programs may and likely will put an alignment gap in the structurally varying region and report the high structural similarity outside this region. During the linear interpolation between the backbones, all points travel the distances scored by RMSD and most coordinate‐based structural alignment scores. In Figure 1, the backbone passes through itself during this implied motion. This self‐intersection is a topological obstruction for a self‐avoiding motion between the two structures and in this case a self‐avoiding motion that untangle the original linear interpolation is significantly longer than reported by traditional structural alignment. The two structures are therefore not close in the configuration space and their folding paths must have branched out at some point. This illustrates why pairs with distinct chain topology can be found relatively independent of structural similarity here measured by global RMSD.

Discrete topological descriptors of protein structures such as knot type and embedding types of lassos, links and θ‐curves detect interesting entanglement of individual structures that challenge our understanding of the folding process. See the review [2]. By the definition of each of these discrete topological descriptors, a direct motion between structures with distinct descriptor values generally makes the backbone to pass through itself and in addition a self‐avoiding path requires a significant untangling motion. As illustration, in [3], the topology was detected changed between one knotted and unknotted structures except for a few cases where the initial structural alignment unties the knot, and a long untangling motion is found. Indeed, protein families with both knotted and unknotted proteins are reported in [4]. Hence there are similar proteins separated by knot type. On the other hand, a change of chain threading as shown in Figure 1 need not, and as we shall see later in this case do not, change knot type, create or delete a lasso, and so forth, but may still require a significantly altered folding path as there is no short self‐avoiding path between the two configurations.

The Călugăreanu‐White‐Fuller theorem [5, 6] states for closed double stranded DNA that the topological invariant linking number between the two strands equals the twist of the helix plus the writhe, which is a real‐valued signed global geometric measure of how coiled a curve is. Local contributions to the linking and writhe integrals can, for example, be used to detect linking between two chains [7] and entangled substructures on one chain [8, 9]. The latter is used to quantify entanglement of native states in [9]. The writhe jumps discontinuously when a curve passes through itself. Levitt [10] used this to detect such events on protein backbones in soft‐atom molecular dynamics simulations. The writhe is changed by all continuous deformations and the writhe jumps of two self‐intersections may cancel out. Therefore, self‐intersections cannot be detected by global writhe values alone. The generalizations of writhe originating in so‐called Gauss‐integral formulations of Vassiliev knot invariants are more powerful at detecting topological variations. A set of 30 of these global descriptors can separate protein folds [11], and all examples of alternate chain topology reported here are expected significantly to change these descriptor values. However global descriptors do not tell where eventual topological changes take place in a pair of structures. Here we apply ProteinAlignmentObstruction [3] that examines the possibility of finding a self‐avoiding path near a structural alignment and provides a mathematically rigorous model of what a change of chain topology is.

Saldaño et al. [12] study apo and holo conformers that generally differ by primarily side‐chain motions, flexible loops in one domain or hinge motions of relative rigid domains. They show that AlphaFold2 predictions prefer one conformer and that predictions worsen with increased conformational diversity. Similarly, AlphaFold2 predictions generally capture one conformer of fold switching proteins [13]. When restricted to domains these conformers are expected to be connected by smaller continuous deformations and therefore not to show up in this study, but these studies emphasize the importance of detecting and keeping structural variation when representative sets of structures are generated.

Homologous but sequence‐dissimilar pairs of CATH domains were relatively often (15%) found to be topologically distinct in [3]. To investigate chain topology of sequence‐similar pairs, we note that sequence‐based structural alignment reveals larger structural dissimilarity than structure‐based does [1] and want the possibility to include a sequence alignment in a structural alignment. Additionally, a topology check of a structural alignments requires a gap‐free structural alignment of the backbone curves [3]. We therefore design BCAlign to include an existing partial alignment of residues (here a sequence alignment), extend it to a gap‐free curve alignment and put the structures in optimal 3D‐superposition. The structural part of BCAlign is based on possibly the only gap‐free alignment algorithm Area‐$C^{\alpha }$ [14] that minimizes the area of a triangulated surface spanned between two protein chains. Several metrics have been used to quantify how different two alignments of the same sequences are [15]. We define the root mean square derivation between sequence alignments. The sequence alignment found by BCAlign minimizes a user‐input convex combination of squared triangle area of a surface spanned between two structures and bounds on the squared derivation between the underlying sequence alignment and the input sequence alignment applicable for dynamic programming [16]. The resulting aligned curves are put in optimal RMSD superposition. The sequence alignment input to BCAlign can instead be a structure‐based sequence alignment provided by a structural alignment program. This enables topology check as a post process to any structural alignment method as was done with TM‐align [17] by simply filling alignment gaps by linear interpolation along the backbone curves in [3]. BCAlign is thus a geometrically improved method to fill alignment gaps before a topology check and a global structural alignment method by itself.

**FIGURE 2:**
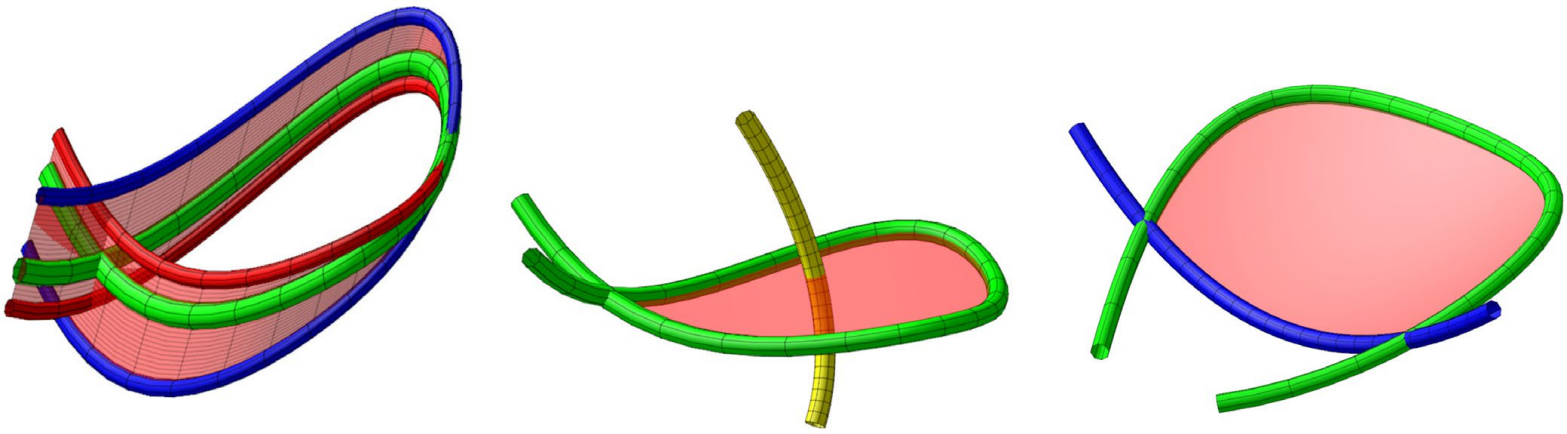
Left: The surface shows the linear interpolation between the red left‐handed loop and the blue right‐handed loop. The curve passes through itself during this motion and the green curve is its configuration at this time of the self‐intersection. Center: Check if the surface spanned by the self‐intersecting arc is disjoint from the remainder of the curve. If the yellow arc is not present then close to the surface and close to the time of the self‐intersection one can before the time of the self‐intersection narrow the loop to have two parallel arcs in the length of the loop, rotate them a half rotation around each other and bring them back to a configuration after the self‐intersection. This motion is called a type 1 move and avoids the self‐intersection found in the original motion. If another arc, like the yellow arc, passes through the spanned surface the type 1 move would cause new self‐intersections and cannot be performed. Right: If two self‐intersections come from the green arc changing between over and under sliding and if the spanned surface is disjoined from the rest of the curve, then the two self‐intersections can be avoided by a type 2 move. Compare [3].

We use ProteinAlignmentObstruction [3] to check the topological similarity of aligned backbone curves. First, it finds steric clashes and all incidents where the backbone passes through it‐self during the linear interpolation between the backbone curves. Some of these morph self‐intersections can be avoided by so‐called type 1 and 2 moves that generalize Reidemeister moves [18] in knot theory [3] and are shown on Figure 2. Here we allow each move to rearrange at most 15 backbone line‐segments and apply the moves that avoid the maximal number of morph self‐intersections at the shortest additional morph‐length. The remaining morph self‐intersections are called essential as a self‐avoiding path requires untangling of too large sub‐structures. Contraction of curve terminals can also be used to avoid morph self‐intersections but are only physically possible when a domain terminal is a chain terminal. A total of 49% of the CATH domains considered here consist of a full chain. We choose therefore not to allow end‐contractions but provide also data from allowing them.

## 2 Materials and Methods

### 2.1 Metrics on Alignments

Let I and J contain the residue numbers of two chains and let $a_{1}:I_{1}↔J_{1}$ and $a_{2}:I_{2}↔J_{2}$ be two alignments defined on subsets $I_{1},I_{2}\subseteq I$ and $J_{1},J_{2}\subseteq J$. Consider first the residues aligned by both alignments $I^{*}=I_{1}\cap I_{2}$ and $J^{*}=J_{1}\cap J_{2}$. Let $\#\left(I^{*}\right)$ denote the number of residues in $I^{*}$ and define the averaged two‐norm ${\text{RMSD}}_{\text{align}}\left(a_{1},a_{2}\right)={\left(\;\frac{1}{2\#\left(I^{*}\right)}\sum_{i\in I^{*}}\left(a_{1}\left(i\right)-a_{2}\left(i\right)\right)2+\frac{1}{2\#\left(J^{*}\right)}\sum_{j\in J^{*}}{\left(a_{1}^{-1}\left(j\right)-a_{2}^{-1}\left(j\right)\right)}^{2}\;\right)}^{1/2}$ and one‐norm, average alignment displacement, $\mathrm{AAD}=\frac{1}{2\#\left(I^{*}\right)}\sum_{i\in I^{*}}|a_{1}\left(i\right)-a_{2}\left(i\right)|+\frac{1}{2\#\left(J^{*}\right)}\sum_{j\in J^{*}}|a_{1}^{-1}\left(j\right)-a_{2}^{-1}\left(j\right)|$. We denote the derivation of an alignment, a, to the exact matching residue pairs in the given sequence alignment, $a_{s}$, by ${\text{RMSD}}_{\text{align}}^{\mathrm{seq}}\left(a\right)={\text{RMSD}}_{\text{align}}\left(a,a_{s}\right)$. Finally, the number of residues not aligned in both alignments called misalignment length MAL measures how unequal the aligned subsets are.

### 2.2 Gap‐Free Structural Alignment and Superposition

**FIGURE 3:**
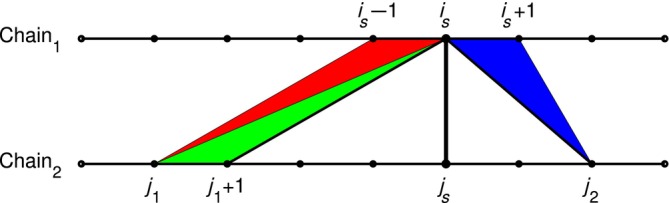
Let $\left(i_{s},j_{s}\right)$ be a sequence‐aligned residue pair and denote the alpha carbons on the first chain by $P_{i}$ and by $Q_{j}$ on the second. The geometric cost for using, for example, the blue triangle is set to ${\left\|\left(P_{i_{s}+1}-P_{i_{s}}\right)\times \left(Q_{j_{2}}-P_{i_{s}}\right)\right\|}_{2}^{2}=4\text{Area}{\left(\left\langle P_{i_{s}},P_{i_{s}+1},Q_{j_{2}}\right\rangle \right)}^{2}$. The red and blue triangles have $i_{s}$ as vertex and bound the part of the second chain that $i_{s}$ can be aligned to compare Supporting Information. The sequence alignment costs of these triangles are $1/2{\left(3.8\;{Å}^{2},\left|\text{bound}-j_{s}\right|\right)}^{2}$. For a consecutively aligned region, the upper and lower bounds equal the final alignment and the costs add up to the square of the area of a rectangle with height 1Å and length equal to the geometric misalignment of residue $i_{s}$. Otherwise, corresponding to alignment gaps, the cost is larger.

BCAlign is described in detail in Supporting Information. First, the structures are put in RMSD superposition [19] of the exact matching pairs and for each chain the window of alignment is from the first to the last exact aligned residue. Consider a triangulated surface connecting the windows of alignment where each triangle, as those shown on Figure 3, has one edge on one of the chains. The core of the algorithm first finds an optimal triangulated surface based on the dynamic programming algorithm from [14] for finding triangulated surfaces with minimum area between two backbone curves. This triangulation is translated into a curve alignment where each alpha carbon is aligned to either an ordinary alpha carbon or to a pseudo alpha carbon added between alpha carbons along the backbone curve. The equal cardinality aligned curves are superimposed in one RMSD call. The geometric and sequence alignment‐based costs of triangles are illustrated on Figure 3 and treated in details in Supporting Information. The triangulation minimizes the linear combination of 4k times the sum of squared triangle areas and $\left(1-k\right)$ times square of $3.8{Å}^{2}{\text{RMSD}}_{\text{align}}^{\mathrm{seq}}\left(a\right)$. The input parameter $k\in \left[0,1\right]$ interpolates between structure‐based alignment for k=1 and sequence‐based structural alignment for k≈0. If the alignment windows are wished fixed, new triangulations and superpositions are found until convergence.

The dynamic programming finds the minimal cost of triangulating any sub‐window with the same starting point and arbitrary terminal point. Hence, one can extend the triangulation to include both C‐terminals and cut the longer chain where the smallest triangulation cost per length is found. The N‐terminal may be treated similarly by reversing the direction of traversal. To get a symmetric algorithm, we near the middle of the alignment windows choose an aligned residue pair with a neighborhood of nearly consecutive aligned residues. Fixing this aligned pair, the full upstream and downstream structures are triangulated and potentially cut in N‐ and C‐terminals, respectively. The two parts are stitched together, re‐parameterized and RMSD superimposed, and the alignment windows are updated. This double‐step is repeated until convergence. If the alignment of a few residues keeps changing periodically, convergence is not reached. The stop criteria for BCAlign used here are maximally 20 iterations and the algorithm stops if the last alignment at most differs by one residue, MAL≤1 and by AAD≤0.015 residues from an alignment from a previous iteration. For 100 aligned residues, AAD=0.015 corresponds to change one alignment to a neighbor residue and another alignment to a neighboring pseudo alpha carbon half a residue away.

### 2.3 Data

We downloaded the CATH4.2 S100 domains and call a chain connected if the next alpha carbon either has a residue number at most three higher than the previous alpha carbon or if their distance is at most 6 Å. We accept repeated and decreasing residue numbers and find 80 628 connected single chain domains after removing a few where upper and lower case chain id's seem confused. Of these, 69 182 take part in 966 546 pairs with at least 30% sequence identity. They represent 957 topologies, 2961 homology families, and 12 549 sequence families. A total of 922 754 pairs are both solved by X‐ray crystallography (a resolution is given in the pdb‐file), 34 190 pairs are X‐ray‐NMR pairs, and 9602 pairs are both NMR structures (no resolution). Sequences alignments are done by the Matlabs implementation of Needleman–Wunsch algorithm [20] with gap opening penalty 8 and gap extension penalty 1 (one) to favor compact alignments at domain terminals. The structural comparison of each pair is performed for 15 values for the parameter $k\in \left[10^{-4},1\right]$. All alignment data and a script making the figures on them are deposited at [21]. We refer to k=0.01 and k=1 as sequence‐based and structure‐based structural alignment, respectively. The number of iterations is highest and on average 3 for low sequence similarity and k=1. Otherwise, the convergence of alignment and superposition is confirmed by the second iteration in 93% (k=0.01) to 82% (k=1) of the alignments. The average computation time is $0.35s\;{\left(\#\text{Residues}/1000\right)}^{2}$ for structural alignment and $0.186s\;{\left(\#\text{Residues}/1000\right)}^{2}$ for the topology check. Cases with topological obstructions have slightly longer alignment times, see Supporting Information.

## 3 Results

### 3.1 Sequence‐Similar RMSD‐Dissimilar Domain Pairs and Alignment Plasticity

**FIGURE 4:**
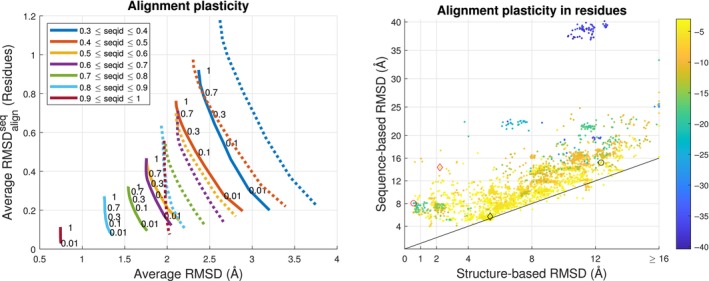
Left: RMSD decreases and ${\text{RMSD}}_{\text{align}}^{\mathrm{seq}}$ increases when k (shown in numbers) is increased. Dotted lines show the 33.3% cases where sequence‐based and pure structural RMSD's differ more than 0.1Å. As expected, structural similarity is higher and structural alignment is closer to sequence alignments for higher sequence identity. Right: 6961 cases where the change in ${\text{RMSD}}_{\text{align}}^{\mathrm{seq}}$ (shown in colors) is ≥3 residues between sequence‐ and structure‐based alignments. The structure‐based RMSD's are relatively uniformly distributed between 1.5 and 14 Å except for the 2408 cases from class 3 30 572 10 1 lying close to the black diamond. The blue cluster in the top of the plot all involve domain 1qu1F00.

There are no identical but many highly similar structures in our data set and sequence‐ and structure‐based RMSD's, and their sequence alignments are almost identical for most pairs. See Figure 4 and Supporting Information. But, there are significant exceptions. We find 12 231 (6315) pairs or 1.27% (0.65%) with sequence‐based (structure‐based) RMSD>6Å. For RMSD>3Å, the similar numbers are 86 178 (56270) pairs or 8.92% (5.82%). Thus, as expected from the study of full chains [1], sequence‐similar RMSD‐dissimilar domains are not uncommon especially for sequence‐based alignments. We align full domains up to eventual cutting of the longest domain terminals. Hence, a smaller structure‐based RMSD is generally found for an alignment with larger ${\text{RMSD}}_{\text{align}}^{\mathrm{seq}}$ to the sequence alignment and not as result of aligning fewer residues. We use ${\text{RMSD}}_{\text{align},k=1}^{\mathrm{seq}}-{\text{RMSD}}_{\text{align},k=0.01}^{\mathrm{seq}}$ to quantify the difference of the two alignments. The 6961 pairs where this difference is larger than three residues are illustrated on Figure 4 (right) and cover 147 T‐classes, 229 H‐classes, and 393 sequence families. Structure pairs with a close structural match for alignment far from their sequence alignment are primarily periodic structures as, for example, the Ankyrin repeat‐containing domain 2y0bH00 that has lost one repeat compared with 3zu7bB00. This pair is indicated by the red circle on Figure 4 (right). The pair (4bflC01, 4qolA00) is indicated with a red diamond on Figure 4. Most of both their sequence‐ and structure‐based alignments are shifted around 70 residues but relatively few residues near the terminals find seemingly random sequence matches with several larger gaps to the opposite 70 residue tails. This causes sequence‐based RMSD to be far greater than structure‐based even if the average change in alignment of the sequence‐aligned pairs, ${\text{RMSD}}_{\text{align}}^{\mathrm{seq}}$, is small. This explains why changes in ${\text{RMSD}}_{\text{align}}^{\mathrm{seq}}$ and in RMSD between sequence‐ and structure‐based alignments, shown in Supporting Information, can be relatively independent. It lies outside the scope of this work to consider if isolated parts of sequence alignments should be ignored but the gap extension penalty in the sequence alignments reduces the number of such cases considerably.

### 3.2 Examples and the Length of an Untangling Motion

**FIGURE 5:**
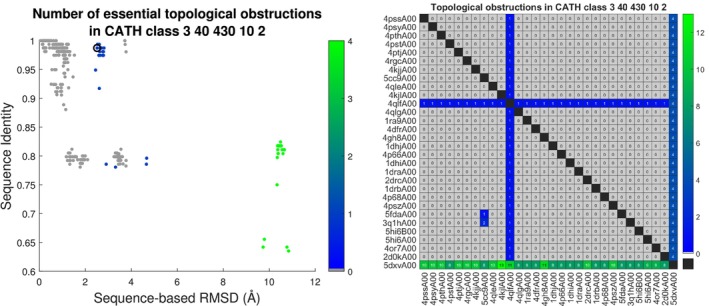
Left: RMSD and sequence identity of all sequence‐based alignments in the sequence family of 4qlfA00 colored by the number of essential self‐intersections. The circle indicates the alignment shown on Figure 1. Right: The number of morph self‐intersections below the diagonal and of the essential ones above.

After the beta strand at the N‐terminal, 4qlfA shown on Figure 1 passes outside an open loop whereas 1ra9A and all other domains in the sequence family pass on the other side. The secondary structures connected by this open loop are not in contact and both structures are therefore lasso free according to the LassoProt server [22]. Hence, the changed chain topology does not create or delete a lasso. The knot type is also unchanged as both structures are unknotted. We note that these topological descriptors of single structures cannot detect the altered chain topology. The A chain of 4qlf is separated by this essential self‐intersection from all other domains in its sequence family, see Figure 5. Residues 122 and 123 are missing from 4qlfA relatively close to the self‐intersection resulting in the long line segment on which the sequence‐based curve alignment inserts two pseudo alpha carbons that receive 122 and 123 as indices in the curve alignment (With resolution 1.44 Å the altered chain topology seems clear from the crystal structure. The example is chosen as it is easy to visualize in one 2D‐projection.) See Figure 5. The designed rethreading 5dxvA [23] is another isolated domain. 5dxvA is separated from the other domains by sequence (structure)‐based RMSD around 10 (7.5) Å and 4 essential morph self‐intersections, see Figure 5. The domains in the sequence family share secondary structure elements but the essential topological obstructions, that all involve 4qlfA or 5dxvA, show that the structures occupy three remote parts in the configuration space.

**FIGURE 6:**
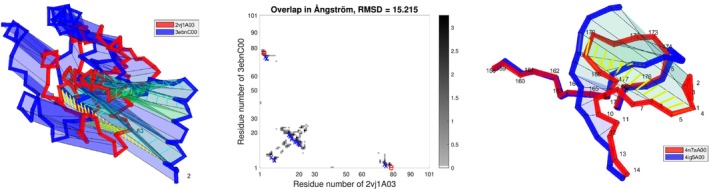
Left: A large motion of the N‐terminal helix from 2vj1A03 to the sequence identical 3ebnC00. Center: Steric clashing during the motion is shown in gray scale and 8 self‐intersections are marked “x” and “e.” The five self‐intersections that occur in the moving helix have indices up to 30 and are resolved. Three self‐intersections are caused by the N‐terminal passing through the chain around residue 80. Two of these can be removed by a type 2 move. The last one cannot, as the chain seen on a larger scale passes through itself once, and is marked “e.” Right: One essential self‐intersection near the N‐terminal of the sequence identical RMSD=2.34Å pair 4n7aA00 and 4ig5A00.

Figure 6 (left and center) shows SARS‐CoV main protease C‐terminal domain that folds significantly different in the domain swapped dimerization, 3ebnC00, than as the third domain in a monomeric chain, 2vj1A03, [24, 25]. The morph between them can be untangled by allowing a move involving 75 of the 101 residues or simply by contracting the first few residues of the N‐terminal. However, the contraction is not practical on the full A‐chain of 2vj1 due to the two domains lying upstream of 2vj1A03. In sequence family 1 10 640 10 3, all morph self‐intersections involve either 4qjqA00 or 4ig5A00 whose N‐terminal lies on the opposite side of the chain around residue 170 than the N‐terminal of all other structures. See Figure 6 (right). Both 4qjq and 4ig5 are obsoleted in the PDB due to paper retraction, are kept in CATH, and serve as an illustrative example. In the case shown on Figure 6 (right), there are three self‐intersections and two are removed by a small type 2 move. The last self‐intersection is indicated as essential on the figure and can only be removed by contracting the N‐terminal. In other alignments within this sequence family, the N‐terminal extends a bit further causing an additional self‐intersection with the chain around residue 174. In these cases, all self‐intersections can be removed by type 2 moves without contracting the N‐terminal. These two examples illustrate why end‐contractions are optional in the program, but even with end‐contractions there will be cases where small perturbations change if topological variation is picked up or not.

There are cases where a significant structural variation does not result in essential self‐intersections but instead in an involved motion untangling all morph self‐intersections. We give the rule of thumb, compare Supporting Information, that untangling motions from 140Å, typically by moving 20 residues 7 Å additionally to the RMSD motion, may be caused by a significant structural variation, for example, the 3.5(3.4) Å RMSD motion of the sequence(structure)‐based structural alignment from 5dwyA00 to 2nwwA00, shown in Supporting Information, has 5(2) self‐intersections caused by swapping two stretches of the chain at the solvent‐accessible surface. The untangling motion of this perhaps significant structural variation is 188(123) Å. When one or two type 2 moves can untangle the morphs similar to 6 (right) in the sequence family of 4n7aA00, they involve 11–21 residues and the untangling motions of 70–150 Å are mostly below this threshold. End‐contractions typically require relatively large motions compared with type 1 and 2 moves, see fig. 4 in [3].

### 3.3 Sequence‐Similar Domain Pairs With Topological Dissimilarity

**FIGURE 7:**
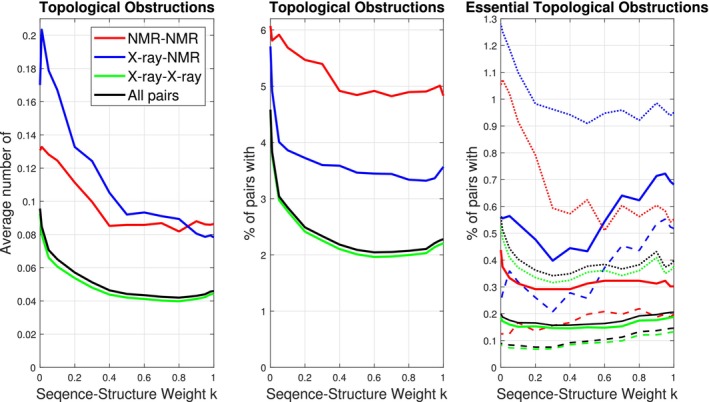
Left and center: Occurrences of topological alignment obstructions. Right: Pairs with an essential self‐intersections (solid line), with an essential self‐intersection when end‐contractions of up to 7.5 residues are allowed (dashed), and with an essential topological obstruction or untangling motion ≥140Å (dotted).

Some domain pairs have significantly different sequence‐based and pure structural alignments. Thus, the topological obstructions to alignments and the ability of type 1 and 2 moves to untangle them may and do as shown on Figure 7 change when the parameter k varies. There are essential morph self‐intersections for at least one value of k for 4386 structure pairs covering 143/215/353 T/H/S classes. Allowing end‐contractions these numbers drop to 2502 structure pairs covering 94/139/215 T/H/S classes. The number of alignments with essential topological obstructions and the number of essential topological obstruction or untangling motion >140 Å are both lowest for k=0.3, where alignments compromise between the given sequence alignment and structural alignment. When measured by RMSD, the larges structural variation is represented by NMR structures, compare Supporting Information, both due to the experimental conditions and as NMR can resolve less stable proteins. We therefore expect and find structural alignments of pairs of NMR structures more frequently obstructed by a morph self‐intersection than alignments of X‐ray‐NMR and X‐ray‐X‐ray structure pairs. Porter and Looger [26] often find one conformation of fold‐switching pairs to be found by noncrystallographic methods and argue that the less stable configuration of fold‐switching pairs are harder to resolve, underrepresented in the PDB and likely therefore also in AlphaFold2 predictions [13]. Similarly, to the frequencies of fold‐switching pairs, we find essential obstructions more frequent between X‐ray and NMR structures than between NMR structures.

**FIGURE 8:**
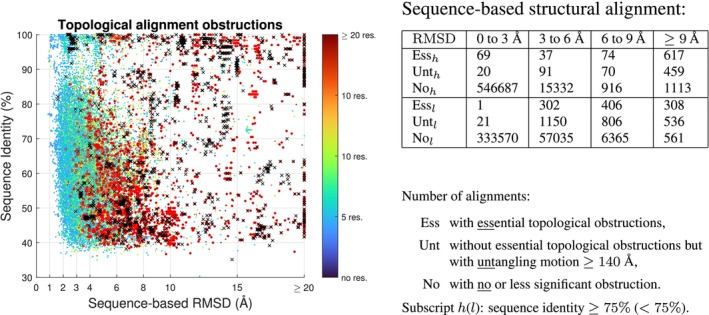
Left: RMSD and sequence identity for 36 992 alignments with topological obstructions. The points are colored by the number of residues involved in untangling the obstructions or shown as a black x if an essential topological obstruction is found. Red dots are cases with untangling motion ≥140Å.

**FIGURE 9:**
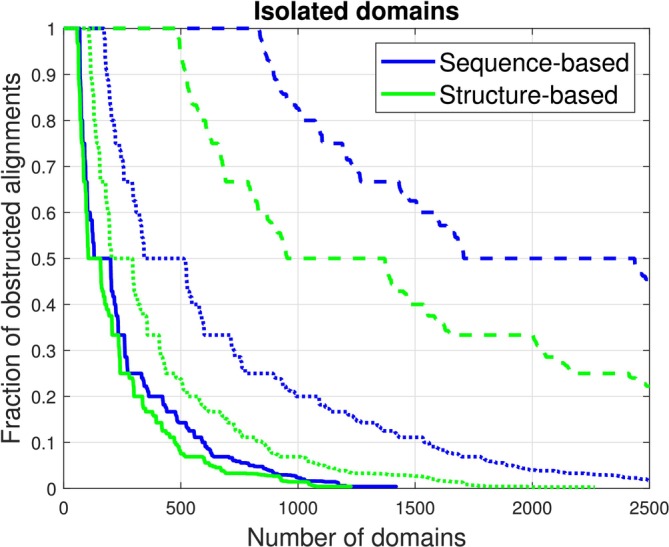
The fraction of obstructed S‐class alignments for each domain is shown in decreasing order. The obstructions are at least one morph self‐intersection (dashed), at least one essential self‐intersection or untangling motion ≥140Å (dotted), and at least one essential self‐intersection (solid line). Four hundred and eighty‐eight domains have self‐intersections in all structure‐based alignments. Five hundred and thirteen domains have either essential topological obstructions or untangling motion ≥140Å to at least half of their sequence‐based alignments.

Figure 8 shows that cases with distinct chain topology detected by at least one essential morph self‐intersection and cases with a significant untangling motion are spread relatively evenly over the range of RMSD and sequence identity. Similar results are found for smaller RMSD‐values for structure‐based alignment and shown in Supporting Information. Several CATH domains are as the above mentioned 4qlfA and 5dxvA topologically distinct from their sequence family, see Figure 9. Using sequence(structure)‐based superposition (and allowing end‐contractions) there are 422(336) [179(182)] domains with essential topological obstruction to at least 20% of the other domains in their sequence family. Counting also untangling length ≥140Å as a significant obstruction, the similar numbers are 1090(578) [920(480)]. A self‐avoiding motion from one structure to another may even in cases with nonessential topological obstructions have to pass through configurations at larger distance than between the two aligned structures. Topological obstructions may therefore be metric barriers for homology modeling and the 2437 (3.5%) domains with topological obstructions to at least 50% of their sequence‐based structural alignment can be challenging independent of sequence similarity.

### 3.4 Larger Motions With Minor Steric Clashing

There are 6219 pairs with both sequence‐ and structure‐based RMSD>6Å. Of these large motions 2204(2590) sequence(structure)‐based structural alignment morphs are self‐avoiding and 985(988) have essential self‐intersections. Of the 3030(2641) pairs with nonessential self‐intersections the untangling motion is small ≤30Å for 541(254) pairs and large >140Å for 843(991) pairs. This gives 1828(1979) cases with significant alignment obstructions but also 2745(2844) cases where at‐most a small correction to the straight line in the configuration space between the backbone curves is self‐avoiding. We can further differentiate how easy the motions are to perform by the average sum of one residues steric clashes with all other residues during the linear interpolation between the structures called the MeanOverlap [3] and shown on Figure 6 (center). Distributions of MeanOverlap values for the large motions are shown in Supporting Information. For self‐avoiding morphs, MeanOverlap starts from 0Å and is only larger than 1Å in 32 cases. The conformational transition of the glutamate transporters between outward and inward facing states, [27], 5cfyA00 and 3kbcA00 has RMSD=10.0Å and only MeanOverlap=0.06Å. Two sub‐structures perform almost rigid rotations. Intra sub‐structure residues do not clash as they perform similar motions but, in this case, also hinge and inter sub‐structure residues have minimal classing. See the animation in Supporting Information in [27]. Cases with high MeanOverlap are generally cases with more involved motions as, for example, the RMSD=16.3 motion from 4fu3A00 to 4fldA00 involving different rotations of individual helices and even breaking and back‐bending a long helix, but there are also several seemingly simpler cases where one 180° rotation of a usually terminal section causes significant morph overlap within that section. See 5iznE00 and 5iznD00 in Supporting Information. Three of the 98 fold‐switching pairs reported in [26] are represented by four pairs of the connected CATH‐domains considered here. The switching between secondary structure types that is the focus of [26] and reported in [28] cause self‐avoiding morphs with some steric clashing. This is expected as the switching is performed by a relative local motion.

## 4 Discussion

In weaker aligned often terminal regions, there are cases where relatively few isolated amino acids are sequence‐aligned seemingly by chance from a structural point of view. These pairs cause sequence‐based structural alignments with larger RMSD and more morph self‐intersections than if these pairs are ignored. Attempts to clean up sequence alignments in weakly aligned regions have led to almost completely ignoring other sequence alignments that are in full agreement with their structural alignment. We have therefore kept the full CATH domains and their full sequence alignments. Instead such structurally challenging sequence aligned pairs are the first to be changed when raising the weight on structural derivation k from 0.01 to, for example, 0.3.

The average resolution value 2.175Å of the topologically isolated X‐ray structures listed in Supporting Information is slightly larger than the average 2.135Å over all the connected X‐ray structures in this study. The worst resolution values for the isolated cases are 3–3.3 Å where the electron density usually allows reliable tracing of the protein backbone, but the identification of side chains start to become challenging [29]. We allow at most two consecutive alpha carbon to be missing or a distance <6Å to the next alpha carbon. Hence, the topology of the curves used here is expected to be in concordance with the electron densities. The slightly lower resolution of the topologically isolated domains may be by chance, but it would be interesting to study if topologically alternate configurations are less stable.

In sequence family 1 20 210 10 1, the domains 2gsmC00, 3omiC00, 3omaC00, and 1m57A00 have sequence‐based RMSD≈3.3Å and one essential topological obstruction to 31 of the 55 other domains due to a larger insertion. Type 1 and 2 moves rearranging 20–25 residues can generally untangle the contraction of the insertions. Such large moves may be reasonable to apply to 530–547 residue chains with insertions as big as 27 residues. However, rearranging 25 residues may significantly alter some folds, especially shorter domains that tend to have shorter secondary structure elements. We therefore only allow untangling moves involving at most 15 backbone line segments each since 76% of the domains considered here have at most 200 residues.

The length of each potential untangling move is calculated, and the sum of these lengths is minimized in the final untangling motion. This sum is an upper bound on untangling motion length and it may seem large in cases with clusters of self‐intersections needing similar untangling motion, compare Supporting Information. Curve smoothing removes local zigzag from backbone curves whereby a cluster of self‐intersections often is reduced to one self‐intersection. The average number of self‐intersections, but not the essential ones, is reduced by a factor of 2.6 [3] by curve smoothening. This makes topology checks easier and improves the estimated untangling motions length. However, without the structural details of the alpha carbon curve, structural alignments based on the area functional tend to drift locally. With focus on plasticity of alignments and the connection to the sequence alignment, we do not apply smoothing here, but mention it as a natural choice in other settings.

Examples of analogous knotted and unknotted proteins with the same function are known [2]. Thus, depending on the location of altered chain topology relative to a protein's active site, its eventual allosteric regulation or functional motion one would expect to find cases both with and without functional consequences of altered chain topology. It is thus interesting to examine if sequence‐similar examples found here combined with the relative frequent examples inside homology and topology classes [3] can explain some apparent structure–function disparities where structures similar by traditional alignment methods have distinct functions [30]. The presented method can help subdividing structural clusters as, for example, in [30], that guarantee many residues in close proximity, into structures with similar chain topology.

## 5 Conclusion

We present a novel method BCAlign for gap‐free structural alignment and superposition of two protein backbone curves needed to quantify steric and topological obstructions to deform one backbone curve into the other. It takes a sequence alignment as input and optimizes a user‐input convex combination of global structural derivation and the derivation of the underlying curve alignment from the input sequence alignment. For this, we introduce RMSD as metric on the space of alignments and also the similar L1‐metric used to guaranty convergence of the sequence alignment underlying a structural comparison.

For sequence‐similar connected CATH domain pairs, we as expected, for example, from [1, 12, 13, 25, 26] find structure‐dissimilar pairs as measured by RMSD. However, relatively independent of structural RMSD and of sequence identity there are pairs with altered chain topology. Structural variation both in terms of RMSD and chain topology is largest for sequence‐based structural alignments that best represent motions of highly sequence‐similar chains. Chain topology is significantly more likely to change between an NMR and an X‐ray structure than between two X‐ray structures and even between two NMR structures that on average are most RMSD‐diverse. On average it is easiest to untangle a path between two aligned and superimposed structures when the alignment optimizes the convex combination of sequence‐ and structure‐based structural alignment given by the parameter k=0.3. This is both the case when counting pairs with essential topological obstructions and when also counting pairs with untangling motions larger than 140Å.

A self‐intersecting backbone motion that changes the chain topology can be of size like a structural change in a flexible loop. Hence the lower RMSD‐cases with altered chain topology are likely to be detected as flexible examples by traditional structural alignment methods. But acknowledging that folding paths have branched out as done here would, for example, rely on careful visual inspection initiated by additional, for example, functional information. We note that the simple notion sequence identity can also not detect altered chain topology and provide a list of more than 25 000 domain pairs with RMSD>5Å or with moderate to essential steric or topological alignment obstructions, for example, to help prediction of alternative configurations. The high RMSD‐cases are naturally most likely to have altered chain topology, but we show that many pairs are connected by larger but easy and in some cases even steric obstruction free motions. We conclude that the topological information enabled here is a significant addition to traditional structural comparison. It allows structural similarity to be correctly inferred at longer distances as altered chain topology can be filtered out or searched depending on the application. We leave it to further studies to examine its importance where structural comparison has been instrumental including structural classification, prediction, model comparison, and biological function for further studies. BCAlign and a few simpler structural alignment methods followed by the topological ProteinAlignmentHindrance check can be run on our server https://stopmh.compute.dtu.dk and a multi‐mer version is in preparation.

## Author Contributions

**Peter Røgen:** software, data curation, methodology, writing – original draft, investigation, visualization.

## Conflicts of Interest

The author declares no conflicts of interest.

### Peer Review

The peer review history for this article is available at https://www.webofscience.com/api/gateway/wos/peer‐review/10.1002/prot.26753.

## Supporting information


Data S1.

## Data Availability

All alignment data and a script making the figures on them are deposited at [21] where also the domain pairs with moderate to essential steric or topological alignment obstructions described above are listed.
