# Supplementary material for: Sequence‐Similar Protein Domain Pairs With Structural or Topological Dissimilarity
Source: Proteins. 2024 Oct 11;93(3):588–97. doi: 10.1002/prot.26753 (PMC11809131; doi:10.1002/prot.26753)
Supplement: Supplementary file 1 — Data S1. [file PROT-93-588-s001.pdf]

Supporting information for

Sequence-Similar Protein Domain Pairs with Structural or Topological Dissimilarity

Peter Røgen
Department of Applied Mathematics and Computer Science,
Technical University of Denmark, Building 303B,
DK-2800 Kongens Lyngby, Denmark
e-mail: prog@dtu.dk

August 9, 2024

1 Materials and Methods

1.1 Variations of the Area-C^α algorithm

The Area Functional with Fit Comparison (AFFC) (1) is a global protein alignment and superposition algorithm. It spans a triangulated surface between two alpha carbon curves and minimizes the surface area. First, AFFC for fixed superposition applies dynamic programming to find a triangulation with minimal surface area. For this triangulation it searches for a better superposition that minimizes the triangulated area, then a new triangulation etc... Falicov and Cohen apply combinations of methods to find a good local minimum at least when similar sub-structures are present. The fact that several local minima are possible is the reason why the mathematical notion minimal surface refers to stationary points of the area functional and not to actual minimizers. In practice the global alignments and superpositions presented in (1) seem to work well but the computation times are not short. The minimal triangulated surface always connects the entire two backbone curves and gives a global structural alignment and superposition. The main reason for doing this (1) is to get a *"physically insightful number that takes insertions and deletions into account"*. At first, it may be surprising that this global match often gives good alignments of similar substructures relatively independent if other parts of the structures are dissimilar. See, e.g., Figure 4 in (1). The following observation at least partially explains this. For similar substructures in meaningful superposition, the backbone curves are roughly parallel. The surface area between them is thus close to the average length of the curve segments times the average distance between them. In random triangulations the directions and positions of the backbone line segments are unrelated. The expected surface areas are only half of those in the parallel case. The optimization finds better than random triangulations of structural dissimilar regions and they thereby contribute even less than the random expectation. By construction, the emphasis is thus highest on finding good alignments of similar substructures. If the backbone curves are smoothened before superposition structural variations tend to give relatively parallel curves, Hereby more emphasis is given to these regions and the final superposition has a larger RMSD than for the alpha carbon curves. There are cases where AFFC finds a good alignment of two similar structures A and A^* and almost re-finds it when aligning the larger structure AB to A^*. However, since the entire AB is aligned to A^* the last part of the sub-alignment of A and A^* gets disturbed as B is aligned to it.

The geometric part of `BCAlign` differs from AFFC in several ways. Firstly, `BCAlign` searches for unbroken alignment windows that do not need to equal the full chains. This addresses the above-mentioned sub-alignment problem and allows the algorithm to identify similar substructures while maintaining gap-free alignments. Further, to get a smooth objective function we minimize the sum of squared triangle areas, as opposed to the triangle areas, in the dynamic programming. We also convert the triangulation of the backbone curves to a structure-based sequence alignment, which apparently isn't done in (1). Finally, for computational speed we apply RMSD superposition based on the constructed alignment. We therefore do not use the same objective function for the alignment and the superposition problem. However, each sub-problem has a unique solution, and the resulting alignment and superposition are often unchanged after the first iteration.

For the readers convenience we include the dynamic programming algorithm from (1) finding triangulated surface with minimum area between two backbone curves. Let P_i, $i = 1, \dots, n$, be the alpha carbon atoms of the first protein structure and Q_j, $j = 1, \dots, m$, those of the second. For $1 \le i \le n-1$ and $1 \le j \le m$ let $\mathrm{Type1_a}(i,j)$ be the (squared) area of triangle $\langle P_i, P_{i+1}, Q_j \rangle$ that progresses along the first chain by including P_{i+1}. Similarly for $1 \le i \le n$ and $1 \le j \le m-1$ let $\mathrm{Type2_a}(i,j)$ be the (squared) area of triangle $\langle Q_j, Q_{j+1}, P_i \rangle$ that includes Q_{j+1} to the triangulation. Computationally we use $\mathrm{Type1_a}(i,j) = \|(P_{i+1} - P_i) \times (Q_j - P_i)\|_2^2 = 4\,\mathrm{Area}(\langle P_i, P_{i+1}, Q_j \rangle)^2$. Recursively build the $n \times m$ matrix $\boldsymbol{A}$ from $\boldsymbol{A}(1,1) = 0$ and

$$\boldsymbol{A}(i,j) = \min\big(\boldsymbol{A}(i-1,j) + \mathrm{Type1_a}(i-1,j), \boldsymbol{A}(i,j-1) + \mathrm{Type2_a}(i,j-1)\big).$$

For each index pair (i,j) the element $\boldsymbol{A}(i,j)$ is the minimal area (minimal sum of squared triangle areas) of a triangulated surface connecting the points $P_1, P_2, \dots, P_i$ to $Q_1, Q_2 \dots, Q_j$. The actual minimizing triangulation is found starting from $\boldsymbol{A}(n,m)$ and moving backwards as follows. At the starting point $SP = (n,m)$ only the two points P_n and Q_m are connected by an edge. Move to the smaller of $\boldsymbol{A}(n-1,m)$ and $\boldsymbol{A}(n,m-1)$ and add the corresponding edge and the triangle of type 1 or 2. Continue until $\boldsymbol{A}(1,1)$ is reached. If another starting point, $SP = (k,h)$, is chosen, then the minimal triangulation connecting $P_1, P_2, \dots, P_k$ to $Q_1, Q_2 \dots, Q_h$ is found.

1.2 Structure-based sequence alignment and superposition

From a triangulation, we construct a curve alignment of the alpha carbon curves needed for the superposition. Start from the N-terminal, set $(i^*, j^*) = (1,1)$ and go through the triangles towards the C-terminal. After the first triangle we reach indices $(i,j) = (2,1)$ or $(1,2)$ and do nothing. The first time both $i > i^*$ and $j > j^*$ we align P_{i^*} and Q_{j^*}. If $(i,j) = (2,2)$ that's all we do, but if, e.g., $(i,j) = (4,2)$ we fill out the curve alignment by adding pseudo alpha carbons on the Q-chain by linear interpolation. Hence, P_2 is aligned to $Q_{1+1/3}$ and P_3 to $Q_{1+2/3}$, where $Q_{1+t} = (1-t) * Q_1 + t * Q_2$. The iteration ends by setting $i^* = i$ and $j^* = j$. The pair P_{i^*} and Q_{j^*} are aligned in the next iteration unless the C-terminal is reached. In this case the C-terminal is treated symmetrically to the N-terminal. Integer valued index pairs correspond to a traditional structure-based sequence alignment except that we do not allow alignment-gaps in both curves simultaneously. At traditional alignment gaps, we have inserted pseudo alpha carbons such that each alpha carbon either is paired with a regular or pseudo alpha carbon. The re-parameterized curves have equal cardinality and are RMSD superimposed in one call. Applying this RMSD superposition to the original structures concludes one iteration. The previous triangulation need not be minimal now and is updated - then a new RMSD superposition etc.

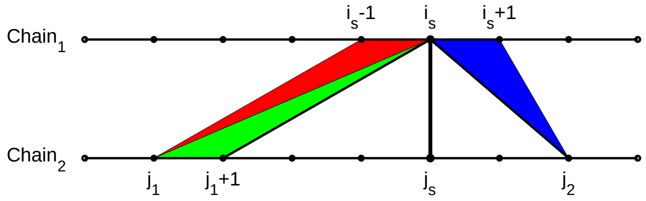

Figure S1: The red triangle $\langle j_1, i_s - 1, i_s\rangle$ has the edge (j_1, i_s) but when followed by the green triangle, residue i_s may be aligned with $j_1 + 1$ indicated by the black line segment. Therefor if $j_1 < j_s$ triangle $\langle j_1, i_s - 1, i_s\rangle$ is penalized by $(j_s - (j_1 + 1))^2$. If $j_s \leq j_1$ residue i_s may be aligned with j_1 and the penalty is $(j_s - j_1)^2$. The penalties for the blue triangle are as for the red triangle after reversing the direction of traversal. This maintains N-to-C symmetry.

1.3 Extending and cutting alignment windows

We initiate our structural alignment with the RMSD superposition of the exact matching sequence-aligned pairs and alignment windows from the first to the last sequence aligned residue on each chain. The aligned windows are not changed by the Area-C$^\alpha$ algorithm and since a better structural alignment or an alignment of larger windows may be obtained by changing the alignment windows we extend the algorithm to enable this. We first describe how to cut alignment windows at their C-terminal. Each element $\boldsymbol{A}(k, h)$ is the minimal sum of squared triangle areas of a triangulation connecting $P_1, P_2, \ldots, P_k$ to $Q_1, Q_2 \ldots, Q_h$. The average squared triangle area $\boldsymbol{A}(k, h)/(k-1+h-1)$ is used to compare triangulations of different alignment lengths. We allow C-terminal cutting of the longest chain down to the length of the shorter chain. The C-terminal cut is done at the residue with minimal floating average of 3 consecutive values of $\boldsymbol{A}(k, h)/(k - 1 + h - 1)$. The floating average helps stabilize the position of the cut when alignment and superposition converge. N-terminal cutting is done as C-terminal cutting but with reversed direction of traversal. We get an N-to-C symmetric algorithm by first calling the original algorithm with the current alignment windows and applying the resulting RMSD superposition to the full chains. Hereafter we near the middle of the alignment windows choose an aligned residue pair with a neighborhood of nearly consecutive aligned residues. Fixing this aligned pair the full upstream and downstream structures are triangulated and potentially cut in N- and C-terminals respectively, stitched together, re-parameterized and RMSD superimposed.

1.4 Combining sequence alignment with the Area-C$^\alpha$ algorithm

We include the squared derivation from the given sequence alignment in the dynamic programming of the Area-C$^\alpha$ algorithm by penalizing triangles violating the sequence alignment. Let (i_s, j_s) be a sequence aligned pair. In a triangulation there are two $\mathrm{Type1}$-triangles with P_{i_s} as vertex. Denote these $\mathrm{Type1}(i_s - 1, j_1)$ and $\mathrm{Type1}(i_s, j_2)$ illustrated in red and blue on Figure S1. Residue i_s is going to be aligned to a residue between j_1 and j_2. To utilize dynamic programming we penalize residue i_s both as being aligned to $j_1 + 1$ and to j_2. Generally $j_1 + 1 = j_2$ in gap free alignments. If $j_1 + 1 \neq j_2$, as shown on Figure S1, the white lower triangle will be filled with $\mathrm{Type2}$ triangles. This triangulation causes i_s to be aligned to $j_s - 1$. The penalties on the endpoints of the interval of possible alignments of i_s are thus larger than i_s's squared alignment displacement. This gives a penalty for having a traditional alignment gap around the sequence aligned pair (i_s, j_s). The

sequence aligned pair $(i_s, j_s) = (4, 3)$ gives rise to the penalty matrix

$$\widetilde{\mathrm{Type1}}_{\mathrm{s}}(4,3) = \begin{bmatrix} 0 & 0 & 0 & 0 & 0 & 0 & \cdots \\ 0 & 0 & 0 & 0 & 0 & 0 & \cdots \\ 1^2 & 0 & 0 & 1^2 & 2^2 & 3^2 & \cdots \\ 2^2 & 1^2 & 0^* & 0 & 1^2 & 2^2 & \cdots \\ 0 & 0 & 0 & 0 & 0 & 0 & \cdots \\ \vdots & \vdots & \vdots & \vdots & \vdots & \vdots & \ddots \end{bmatrix}.$$

The sum of $\frac{1}{2}(3.8\mathring{A}^2)^2\widetilde{\mathrm{Type1}}_{\mathrm{s}}(i_s, j_s)$ for all exact sequence pairs gives $\mathrm{Type1}_{\mathrm{esp}}$ that penalizes the squared distance along the backbone caused by the j-derivations from the sequence alignment. The multiplicative constant $\frac{1}{2}(3.8\mathring{A})^2$ makes $\mathrm{Type1}_{\mathrm{esp}}$ numerically similar to $\mathrm{Type1}_{\mathrm{a}}$ around the sequence alignment. We similarly build $\mathrm{Type2}_{\mathrm{esp}}$ to penalize the squared i-derivations from the sequence alignment. We also build matrices, denoted $\mathrm{Type1}_{\mathrm{oap}}$ and $\mathrm{Type2}_{\mathrm{oap}}$, for the otherwise aligned pairs. All $\mathrm{Type1}$ triangle data is collected in

$$\mathrm{Type1} = k\,\mathrm{Type1}_{\mathrm{a}} + (1-k)\big(\mathrm{Type1}_{\mathrm{esp}} + 0.3 * \mathrm{Type1}_{\mathrm{oap}}\big)$$

and the similarly for $\mathrm{Type2}$. After replacing $\mathrm{Type1}_{\mathrm{a}}$ and $\mathrm{Type2}_{\mathrm{a}}$ with $\mathrm{Type1}$ and $\mathrm{Type2}$ the dynamic program minimizes a linear combination of squared structural deviation ($k = 1$) and squared alignment deviation ($k = 0$).

1.5 Computation time

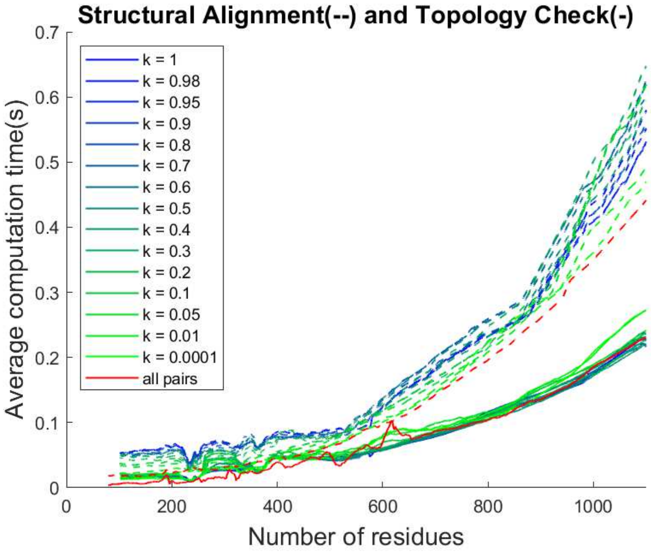

Figure S2: For each value of k the running mean of computation time for alignment (dashed) and for topology check (solid) are shown in blue to green for the 2-4% cases with topological obstructions. In red, the same for all alignments and averaged over k. There are relatively few long chains.

The averaged computation time as function of the number of residues in the alignments is shown in Figure S2. In red the computation times for all aligned pairs. Each iteration of the structural alignment is quadratic in the number of pseudo-residues and since the average number of iterations is

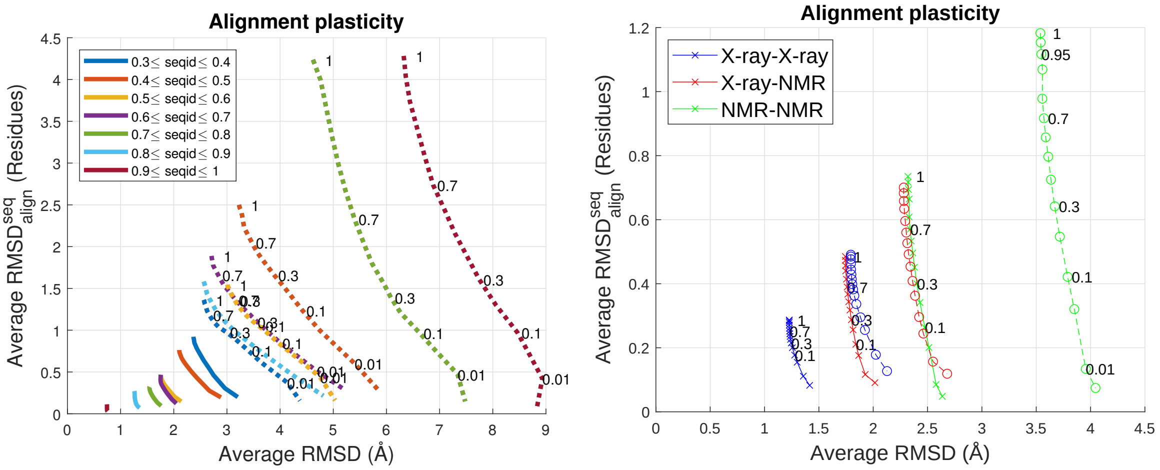

Figure S3: Left: RMSD decreases and $\mathrm{RMSD}^{\mathrm{seq}}_{\mathrm{align}}$ increases when k (shown in numbers) is increased. Dotted the 4.3% cases where sequence-based and pure structural RMSD's differ more than $1\mathring{A}$. Note the large motions of the sequence-similar of these pairs. Right: average $\mathrm{RMSD}^{\mathrm{seq}}_{\mathrm{align}}$ to the exact sequence alignment and RMSD as functions of the parameter k. Markers 'x' for all pairs and 'o' for the 56%, 68%, and 62% of the X-ray-X-ray, X-ray-NMR and NMR-NMR pairs respectively where RMSD changes when k changes from 1 to 0.01.

2.09(2.29) for sequence(structure)-based alignments the general average is quadratic in the number of residues. The topology check consists for most alignments only of calculating the steric clashing during the linear interpolation, which is quadratic in the number of pseudo-residues. When topological obstructions are found the topology check has worse calculational complexity, but in practice calculation time is only slightly raised. In these more difficult alignments with topological alignments obstructions especially the structure-based structural alignment requires more iterations and gets slower. To gain speed the strict criteria on convergence of the sequence alignment underlying structural alignment may be loosened if the focus mainly is on structural similarity and not on the underlying alignments.

In an early stage in this project the objective function for the triangulations was in one-norm, hence a linear combination of the sum of triangle areas and the one norm in sequence space. There were examples that convergence wasn't reached as two or three alignments ended up being repeated periodically. Switching to the sum of triangle areas squared, that is quite similar to RMSD^2 used for the superposition, fewer iterations are needed for convergence.

1.6 Changes in alignment between sequence-based and structure-based structural alignments

The average displacement of the alignment from the sequence-based (structure-based) structural alignment to the given sequence alignment is $\mathrm{AAD} = 0.021$ (0.095)residues corresponding, e.g., to changing 2 (9) out of 100 sequence aligned residues to a neighbor residue. The RMSD-change between the two alignments is $\leq 0.2\mathring{A}$ in 78% of the pairs and for 74% also the AAD-change is ≤ 0.1. Figure S3 shows that if sequence- and structure-based RMSD differ more than $1\mathring{A}$ then sequence-similar pairs especially are highly structurally different. Also fully as expected some structural variation is found between X-ray and NMR structures and more between NMR structures. For many alignments the sequence-based and structure-based alignments are identical. Figure S4 shows that when this not is the case the variation in the $\mathrm{RMSD}^{\mathrm{seq}}_{\mathrm{align}}$ to the sequence alignment and

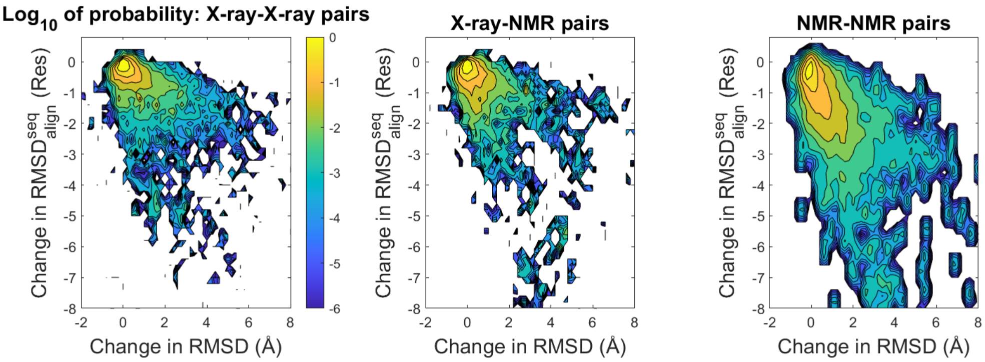

Figure S4: For the 56%, 68%, and 62% of the X-ray-X-ray, X-ray-NMR and NMR-NMR pairs respectively where RMSD changes when k change from 1 to 0.01 are shown the distribution of changes in RMSD and $\mathrm{RMSD}^{\mathrm{seq}}_{\mathrm{align}}$ between structural and sequence-based structural alignment.

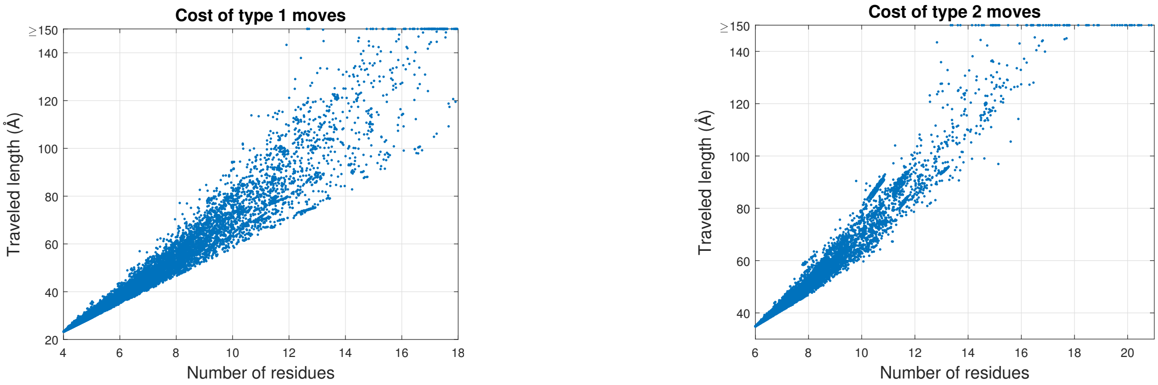

Figure S5: The length of the motion needed to perform type 1 and 2 moves.

in the structural RMSD are relatively independent.

1.7 On the length of untangling motions

Consider a type 1 move untangling a self-intersection between two points lying x line-segments apart along the backbone. The move is assumed to involve $x + 3$ residues as it includes the residue ending the line segments and the two residues neighboring the involved segment. Similarly, a type 2 move untangling two self-intersections by moving two stretches of the backbone, combined containing x line segments, involves 6 additional residues, 3 on each of the two stretches of the chain.

The one self-intersection between 5l8rL00 and 2wscL00 is between points 12.6-line segments apart along the backbone curve. The type 1 move untangling it, involves 15.6 residues moving 152Å additionally to the 4.77Å RMSD. As the involved loop is very open, the untangling motion is one of the longest type 1 moves involving around 15.6 residues, see Figure S5. We find that the length of the untangling motions better captures structural dissimilarity than the number of residues involved in these motions. Figure S6 shows to the right a part of the alignment of 1sg1X01 and 3bukC01 where the first 20 residues perform a half rotation. The linear interpolation collapses in on itself and causes 5(3) self-intersections in the sequence(structure)-based structural alignment. Untangling these involves 23(22) residues with total motion 153(141)Å. In this case several residues take part

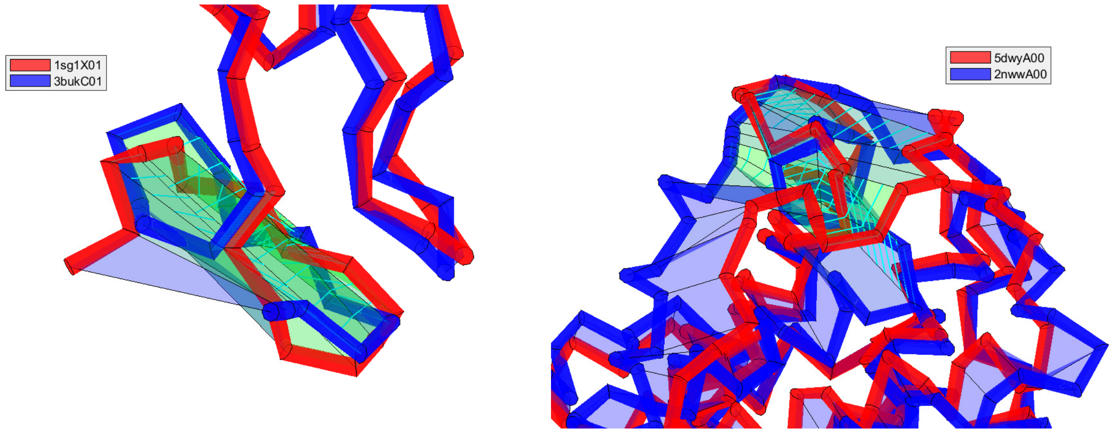

Figure S6: Left: A 180-degree rotation of the N-terminal. Right: When the top front stretch of 5dwyA00 moves to the back top stretch of 2nwwA00 it makes several intersections with the part of the structures sticking out in between them.

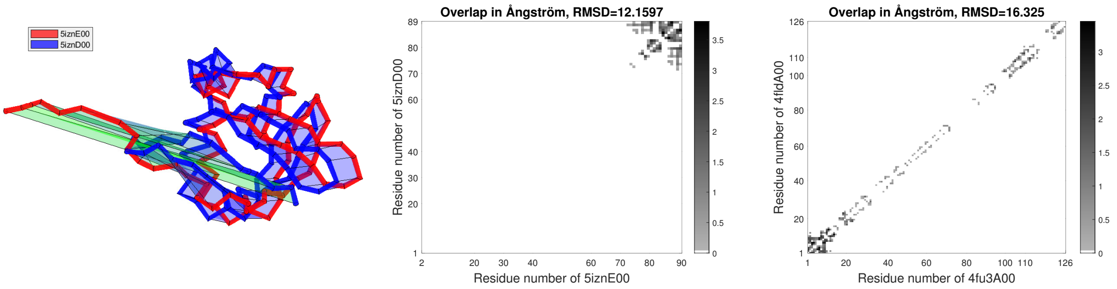

Figure S7: Left and center: Sequence identical domains 5iznE00 and 5iznD00. The C-terminal tail is rotated close to 180 degrees casing significant steric clashing between the involved residues, $1.03\AA$ on average. Right: Steric clashing between 4fu3A00 and sequence identical 4fldA00.

in more than one untangling move. It is only checked if each untangling move can be performed by itself as investigating if one untangling motion can help performing another move at the same time is much more involved. Thus, the lengths of the untangling moves are added as they are performed independent of each other. The estimated untangling motions may therefore seem large in cases with clusters of self-intersections needing similar untangling motion. The untangling of self-intersections occurring in a contraction of a large sequence alignment gap may become $> 140\AA$. Such a case may be considered a false positive to the $> 140\AA$ rule of thumb but as it is to be expected from the sequence alignment it can easily be sorted out automatically.

1.8 Large continuous deformations with little steric clashing

`ProteinAlignmentObstruction` finds the shortest distance between each pair of aligned alpha carbons during the linear interpolation between the two superimposed structures and notes how much it eventually violates a distance constraint derived from a representative set of structures (2). For residues at least 8 residues apart along the backbone the shortest distance is $3.7\AA$. See Figure S7. The $\mathrm{MeanOverlap}$ is the average sum of these steric clashes between one residue and all other residues during the liner interpolation between two aligned and superimposed structures. Figure S8 shows the distribution of steric clashing for structurally dissimilar domain pairs. For

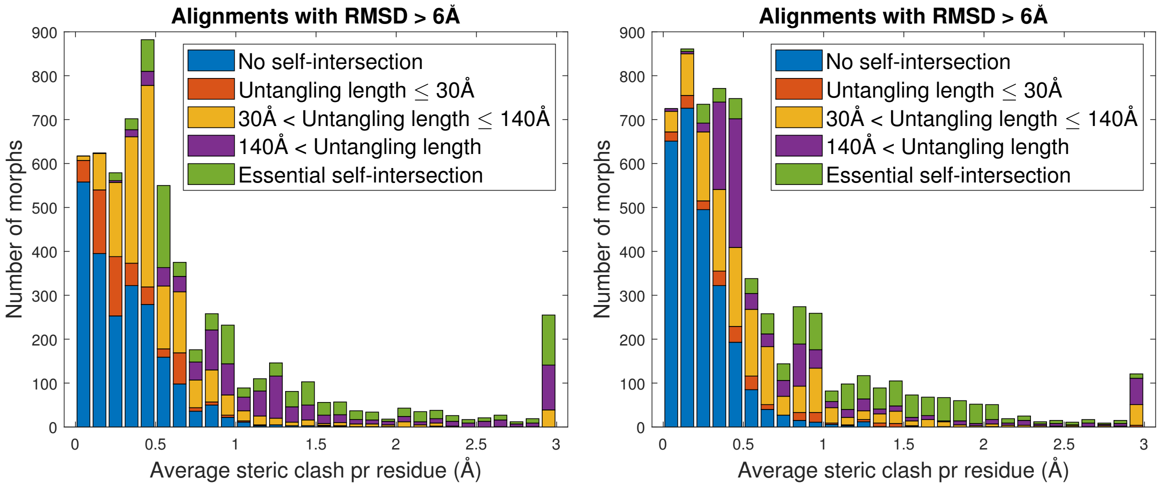

Figure S8: There are 6219 pairs with both sequence- and structure-based $\mathrm{RMSD} > 6\AA$. The distributions of steric clashing during the linear interpolation between the sequence-based (left) and structure-based (right) structural aligned pairs. The cases with non-essential self-intersections are subdivided according to the additional morph length required to untangle the self-intersections.

self-intersection free morphs $\mathrm{MeanOverlap}$ ranges from $0\AA$ to around $1\AA$ quantifying how easy it is to realize the linear motion. If a substructure undergoes a large collective motion then the translational part of the motion will not cause steric clashing as neighboring residues undergo parallel motions, see Figure S7. Linear interpolation between rigid rotated substructures will compress the substructure and cause steric clashing for larger rotations, see the C-terminal on Figure S7. For large rotations of substructures $\mathrm{MeanOverlap}$ grows significantly and self-intersections as, e.g., is the case on Figure S6(left) may appear. This is illustrated by two examples with high $\mathrm{MeanOverlap}$, shown on Figure S7. The first example is 5iznE00 and 5iznD00 where the C-terminal tail is rotated close to 180 degrees causing significant steric clashing between the involved residues, $1.03\AA$ on average. The induced translation of the other part of the structure does not cause steric clashing. The other example is a much more involved motion that also has $\mathrm{MeanOverlap}$ $1.03\AA$. It is the self-intersecting free $\mathrm{RMSD} = 16.3\AA$ sequence-based structural alignment of 4fu3A00 and sequence identical 4fldA00. Several helices are rotated 90 degrees between the two structures and a long helix is broken and bends back on itself. The motion is too involved to be shown in one planar projection but when rotating the aligned structures one can by eye follow the motions of individual parts and realize that they share chain topology. This also follows from the absence of self-intersections and that steric clashing mostly occurs in a narrow band around the diagonal. At the other end of the scale is the pair 5cfyA00 and 3kbcA00 described in the main paper.

1.9 Topological alignment obstructions

We did each of the 966546 domain-pair alignments for 15 values of k steering the relative weight on sequence-based and structure-based structural alignment. Figure S9 illustrates the 26179 alignments with essential topological obstructions to the left. Note that some pairs have essential self-intersections for several k-values and may be represented by several points if these alignments have different RMSD and $\mathrm{RMSD}^{\mathrm{seq}}_{\mathrm{align}}$.

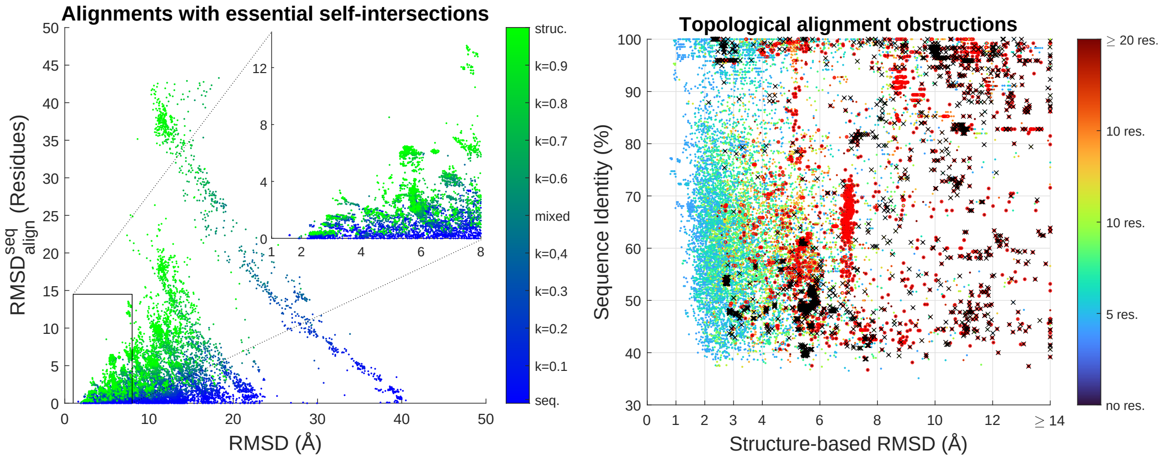

Figure S9: Left: The 26179 alignments and superpositions with essential topological obstructions color-coded by the value of k. If RMSD and $\mathrm{RMSD}^{\mathrm{seq}}_{\mathrm{align}}$ are unchanged for more values of k the highest k-value is shown. Right: The same figure as Figure 8 in the main document, but for structure-based alignments. RMSD and sequence identity for alignments and superpositions with topological obstructions. The points are colored by the number of residues involved in untangling the obstructions or shown as a black x if an essential topological obstruction is found. Red dots are cases with untangling motion $\geq 140\mathring{A}$.

1.10 Table with isolated domains when allowing end-contractions and supporting data

Tables S1 and S2 list the 81 domains with essential topological obstruction to at least half of the other domains in their sequence family when additionally to type 1 and 2 moves also end-contractions of up to 7.5 residues may be applied to avoid morph self-intersections. We list all domain pairs with either larger structural variation, $\mathrm{RMSD} > 5\mathring{A}$, or at least one essential topological alignment obstruction, or larger untangling motion $> 100\mathring{A}$, or larger steric clashing, mean overlap $> 1\mathring{A}$, when interpolated based on the sequence-based structural alignment. The 25719 cases found when also allowing end-contraction to resolve the topological obstructions are found in `SupportingDataEndContractions.xls` and the 25833 cases when only allowing type 1 and 2 moves to resolve the topological obstructions are found in `SupportingDataNoEndContractions.xls`. The full data on all alignments with and without end-contractions are found in `matlabEnd.mat` and `matlabNoEnd.mat` respectively. All alignment data and a script making the figures on them is deposited at Doi: 10.11583/DTU.25442134 Individual alignments can be performed on the server https://stopmh.compute.dtu.dk.

1.11 A Passing problem

The domain 4cuyF00 from class 1 10 455 10 1 is listed as having 206 residues but the file provided holds the 64 residues 89-152. The chain provided is the lowercase f-chain and should have been the uppercase F-chain. There are other such ribosomal examples. In all we removed 17 domains where the number of residues we find differs by more than 3 from what CATH counts. We accept decreasing residue numbers as e.g. found in `1jpl`.

	N	Ess_a	Ess_b	$Obst_a$	$Obst_b$	$RMSD_a$	$RMSD_b$	C	A	T	H	S	$nRes$	Res	seq_b
3czoA01	19	0	0.53	0	6.68	1.73	6.34	1	10	275	10	6	191	2.2	0.43
3hj5B00	36	0.19	0.56	2.69	32.11	9.25	21.46	1	10	790	10	2	102	3.1	0.92
1i4mA00	36	0.89	0.58	6.97	22.44	11.48	20.85	1	10	790	10	2	108	2	0.91
2lsbA00	1	1	1	8	7	13.27	16.77	1	10	790	10	5	142	999	0.87
2prp000	1	1	1	8	7	13.27	16.77	1	10	790	10	5	142	1000	0.87
2lz1A00	2	0.5	0.5	6	4	11.02	16.66	1	10	880	10	1	90	999	0.7
2kz5A00	2	0.5	0.5	6	3	8.81	14.51	1	10	880	10	1	91	999	0.7
2idoB00	1	1	1	2	2	9.1	10.64	1	20	58	250	1	75	2.1	0.56
1du2A00	1	1	1	2	2	9.1	10.64	1	20	58	250	1	76	999	0.56
2pbiA04	2	0	0.5	2.5	17	15.98	23.57	1	20	58	1850	4	156	1.95	0.47
2es0A00	2	0	0.5	1	12.5	10.36	15.41	1	20	58	1850	4	129	2.1	0.47
2gsmC00	58	0.74	0.53	1.74	2.95	2.55	3.09	1	20	210	10	1	534	2	0.54
3omiC00	58	0.76	0.53	1.98	2.84	2.59	3.12	1	20	210	10	1	530	2.15	0.53
3omaC00	58	0.74	0.53	1.98	3.16	2.58	3.1	1	20	210	10	1	531	2.3	0.53
1m57A00	58	0.83	0.53	1.95	2.64	2.61	3.14	1	20	210	10	1	547	3	0.54
4phqC00	2	0	0.5	2	32.5	10.73	17.12	1	20	1170	10	1	298	1.94	0.99
2wcdA00	2	0.5	0.5	4	67	22.89	33.24	1	20	1170	10	1	285	3.29	0.99
1lk3A00	4	0.75	0.75	5	2.5	12.88	18.22	1	20	1250	10	3	136	1.91	0.91
4rzpA00	8	0	0.5	0	3	2.19	5.97	1	25	10	10	19	242	2.8	0.51
4d49A00	4	0	0.75	0	17.5	2.73	7.3	1	25	10	10	47	239	2.09	0.64
4db8A00	4	0	0.5	0	18.5	4.11	13.62	1	25	10	10	47	247	2.5	0.57
1j2oA00	2	1	1	6	6	8.51	13.16	2	10	110	10	28	114	999	0.65
1m3vA00	2	0.5	0.5	2	5.5	8.17	13.1	2	10	110	10	28	122	999	0.73
2lxdA00	2	0.5	0.5	6	5.5	9.89	18.19	2	10	110	10	28	123	999	0.57
2xdpA01	4	0.5	0.5	1.5	1.75	11.61	16.77	2	30	30	140	13	65	1.56	0.60
3gnfB02	1	0	1	1	4	4.85	9.97	2	30	30	560	2	62	2.1	0.94
2zuoA02	1	0	1	1	4	4.85	9.97	2	30	30	560	2	54	1000	0.94
2cwaA01	1	0	1	0	6	3.68	15.03	2	40	50	140	11	107	1.96	0.47
3udgB01	1	0	1	0	6	3.68	15.03	2	40	50	140	11	107	2.4	0.47
1ripA00	1	1	1	2	2	5.59	6.8	2	40	50	140	208	81	999	0.53
1vs7Q00	1	1	1	2	2	5.59	6.8	2	40	50	140	208	81	1000	0.53
1a57A00	5	0	0.6	1.4	1.2	6.12	6.92	2	40	128	20	21	116	999	0.99
5bv2Q01	5	0	0.6	0	19.6	2.02	12	2	40	180	10	3	486	1.53	0.42
4bflC01	5	0	0.6	0	19.2	2.02	12.03	2	40	180	10	3	483	1.64	0.42
3fn5B00	4	0.75	0.5	10.5	6.75	10.84	17.36	2	40	260	10	2	163	1.5	0.64
3fn6A00	4	0.75	0.75	9.5	4.5	10.56	17.87	2	40	260	10	2	174	1.9	0.63
4o8lC00	4	0.5	0.5	6	1.75	8.22	12.63	2	40	260	10	2	173	2.7	0.63
4o8lB00	4	0.5	0.5	6.75	4	9.01	13.35	2	40	260	10	2	155	2.7	0.64
1zk9A00	12	0.5	0.5	4.58	4.42	8.83	11.79	2	60	40	10	149	110	2.18	0.65
1zkaA00	12	0.42	0.5	5.33	3	9.36	13.46	2	60	40	10	149	110	2.2	0.65
3juzA00	12	0.67	0.67	4.83	1.67	8.62	12.01	2	60	40	10	149	101	2.51	0.73
3jssA00	12	0.67	0.67	4.92	1.67	8.59	11.99	2	60	40	10	149	101	2.6	0.74
3jv0A00	12	0.67	0.67	4.08	1.67	8.63	11.95	2	60	40	10	149	101	2.65	0.73
2otpA02	4	0.75	0.5	5.25	14.25	11.7	15.15	2	60	40	10	254	100	2.6	0.47
3jybA00	2	0.5	0.5	3.5	6	5.49	7.75	2	60	40	2380	1	135	2.04	1
3jybB00	2	0.5	0.5	4	5	5.5	7.73	2	60	40	2380	1	135	2.04	1
2xbzB00	2	1	1	7.5	11	10.55	15.04	2	60	40	2380	1	135	2.65	1
4r4xA03	9	0	0.89	0.89	0.89	5.51	6.67	2	60	120	230	4	170	1.9	0.46
4r4zD03	9	0	0.89	0.89	0.89	4.86	6.22	2	60	120	230	4	165	2.81	0.47
1yiqA01	2	0	0.5	0	4.5	2.5	5.95	2	140	10	10	2	578	2.2	0.40
1flgA00	2	0	0.5	0.5	6.5	3.76	5.84	2	140	10	10	2	582	2.6	0.40
2mj3A00	7	1	1	1	2.14	6.82	8.33	3	10	20	30	10	116	999	0.54
1uh6A00	1	1	1	2	18	11.35	16.49	3	10	20	90	139	100	999	0.44
1v9jA00	1	1	1	2	18	11.35	16.49	3	10	20	90	139	113	999	0.44
2octA00	5	0.2	0.6	4.8	5.6	12.55	16.35	3	10	450	10	2	97	1.4	0.83
3qrdB00	8	0.5	0.63	3	15.88	10.69	16.49	3	10	450	10	6	104	2.19	0.68
1cewI00	8	0.25	0.5	2.5	9.5	7.43	11.58	3	10	450	10	6	108	2	0.45
3q94A00	2	1	0.5	4.5	2	4.98	5.83	3	20	20	70	13	276	2.3	0.52
2fjkA00	2	0.5	0.5	2.5	2	3.97	4.28	3	20	20	70	13	297	2.2	0.52
2wskA02	2	0	0.5	0	6	3.76	5.15	3	20	20	80	194	439	2.25	0.48
4j7rA02	2	0	0.5	0	8	5.89	7.7	3	20	20	80	194	514	2.3	0.48
2atcB01	6	0.83	0.83	5	4.67	4.46	5.72	3	30	70	140	1	100	3	0.81

Table S1: When end-contractions of up to 7.5 residues may be applied to avoid morph self-intersections of sequence-based structural alignments, 81 domains have essential topological obstructions to at least half of their sequence family. For these domains we include: the number of alignments of the domain, N, the fraction these with essential topological obstructions to the structure/sequence-based alignments, Ess_a and Ess_b respectively, the average number of topological obstructions, $Obst_a$/$Obst_b$, the average RMSD, the CATHS class, number for residues, $nRes$, the resolution, Res, and the average sequence identity for domains with essential topological obstructions to the sequence alignment, seq_b.

	N	Ess_a	Ess_b	$Obst_a$	$Obst_b$	$RMSD_a$	$RMSD_b$	C	A	T	H	S	$nRes$	Res	seq_b
2kx6A00	4	0	0.75	1.25	3.25	7.91	9.8	3	30	450	20	3	125	999	1
3zxqB00	2	1	1	8	48.5	10.49	14.35	3	30	565	10	30	124	1.9	0.58
3zxoA00	2	0.5	0.5	4.5	24.5	5.99	7.81	3	30	565	10	30	122	1.9	0.58
3zxoB00	2	0.5	0.5	3.5	24	5.93	7.93	3	30	565	10	30	122	1.9	0.58
4lniA02	2	0	0.5	0	3.5	4.22	6.6	3	30	590	10	2	337	2.58	0.46
1f52A01	2	0	0.5	0	2.5	2.84	4.07	3	30	590	10	2	353	2.49	0.46
2v64H00	5	0	1	1.6	6	8.73	9.55	3	30	900	10	2	181	2.9	0.98
1pf4A02	34	0.18	0.97	7.79	23.29	11.2	16.8	3	40	50	300	50	247	1000	0.45
3nv7A00	6	1	1	5	7.5	11.08	19.78	3	40	50	620	19	155	1.75	0.55
4qlfA00	29	1	1	1.17	1.34	2.98	3.1	3	40	430	10	2	157	1.44	0.95
5dxvA00	29	1	1	5.14	9.07	7.47	10.42	3	40	430	10	2	148	1.55	0.79
5ji5A00	6	0	0.5	0	7.33	2.71	7.66	3	40	800	20	4	305	2.1	0.40
3wraA01	2	0	0.5	7	5	4.94	5.41	3	40	830	10	3	303	2.1	0.40
3wrbA01	2	0	0.5	7	3	4.94	5.24	3	40	830	10	3	303	2.1	0.39
1b4uB00	2	0	1	14	8	9.69	10.46	3	40	830	10	3	298	2.2	0.39
4u06A00	4	0	0.5	0	1.25	3.67	6.05	3	80	10	10	7	343	1.9	0.61
3e6jA00	4	0	0.75	0	23	3.32	9.99	3	80	10	10	23	219	1.67	0.49
1qu1F00	69	0.96	0.75	10.67	22.99	11.53	38.59	3	90	20	10	1	155	1.9	0.63
5ac3A00	3	0	0.67	0	7.33	3.25	6.91	3	90	1300	10	1	490	1.8	0.40

Table S2: Continuation of Table S1

References

[1] Alexis Falicov and Fred E. Cohen. A surface of minimum area metric for the structural comparison of proteins. *Journal of Molecular Biology*, 258(5):871–892, 1996.

[2] Peter Røgen. Quantifying steric hindrance and topological obstruction to protein structure superposition. *Algorithms for Molecular Biology*, 16(1):1, 2021.
